# Blood biomarkers for diagnosis and differential diagnosis of Alzheimer's disease in real-world clinical populations: A systematic review

**DOI:** 10.1177/13872877251408510

**Published:** 2025-12-29

**Authors:** Shivani Suresh, Luciana Maffei, Sarah Bauermeister, Vanessa Raymont

**Affiliations:** 1 Department of Psychiatry, University of Oxford, Oxford, UK; 2 Dementias Platform UK, Oxford, UK

**Keywords:** Alzheimer's disease, blood biomarkers, clinical settings, diagnosis, GFAP, NfL, pTau217

## Abstract

**Background:**

Gold standard diagnosis of Alzheimer's disease (AD) relies on invasive, expensive, and non-scalable methods (cerebrospinal fluid lumbar puncture and amyloid-positron emission tomography). Blood biomarkers present a scalable, accessible, and resource-efficient diagnostic alternative.

**Objective:**

To investigate the diagnostic and differential diagnostic performance of three clinically relevant plasma biomarkers: phosphorylated tau-217 (pTau217), glial fibrillary acidic protein (GFAP), and neurofilament light chain (NfL) for biologically confirmed AD patients in real-world, clinical settings.

**Methods:**

A systematic search was conducted across 5 databases for peer-reviewed studies between January 2019-January 2025. A narrative synthesis was conducted for eligible studies.

**Results:**

13 studies (n = 4686 participants) were included. All studies were cross-sectional, and investigated populations recruited from memory clinics, neurology departments, or clinical cohorts. Diagnostic performance of pTau217 was consistently high (AUC > 0.90 across all comparisons). GFAP showed stronger and more consistent diagnostic performance as compared to NfL, though both demonstrated moderate and variable accuracy (AUCs ranging from <0.75 to >0.90). No studies assessed combinations of all 3 biomarkers. Methodological and assay heterogeneity was common.

**Conclusions:**

Plasma pTau217 demonstrated strong diagnostic accuracy and promise for diagnosis of AD. GFAP and NfL displayed inconsistent results, but could provide complementary information, particularly for differential diagnosis. Further standardized studies in underrepresented populations are required to validate and enable blood biomarker implementation in clinical settings.

## Introduction

Gold standard diagnostic techniques for Alzheimer's disease (AD), like positron emission tomography (PET) neuroimaging and cerebrospinal fluid (CSF) lumbar puncture, are invasive, expensive and time intensive. Consequently, their use is limited to high-income countries and specialist tertiary-care clinics, but rare in low- and middle-income countries (LMICs) and community-based clinics, which often lack advanced healthcare infrastructure.^1^ This highlights the need for more cost-effective and scalable alternatives to facilitate timely and accurate diagnoses across diverse clinical settings.^2^

Recent advancements in highly sensitive detection methods have enabled accurate detection of relevant biomarkers in peripheral biofluids such as blood. This is a timely development, as the approval of anti-amyloid therapies for AD, aducanumab in 2021 and lecanemab in 2023, has underscored the need for early and accurate diagnoses, and subsequent streamlining of patients into the appropriate clinical trial.^3^

The clinical promise of AD blood biomarkers is highlighted in the National Institute on Aging-Alzheimer's Association's (NIA-AA) most recent guidelines, “*Revised criteria for diagnosis and staging of Alzheimer's disease*”.^4,5^ Updating the 2018 ATN Research Framework (key differences summarized in Table 1),^5^ this document points to the imminent clinical applicability of blood biomarkers, particularly those that are likely to receive regulatory approval. These are phosphorylated tau-217 (pTau217), glial fibrillary acidic protein (GFAP), and neurofilament light chain (NfL).^4^

**Table 1.** Differences in biomarker categorization between NIA-AA Research Framework (2018) and Clinical Criteria for Staging and Diagnosis (2024).

	Category name	Sub-category	Associated Biomarkers	
			CSF or Plasma	Imaging
2018 Biomarker Categorization	A	/	CSF Aβ_42_, or Aβ_42_/Aβ_40_ ratio	Amyloid PET
	T	/	CSF phosphorylated tau	Tau PET
	N	/	CSF total tau	Anatomic MRI FDG PET
2024 Biomarker Categorization	A	Core biomarkers: Core 1	Aβ_42_	Amyloid PET
	T1		p-tau 217, p-tau 181, p-tau 231	/
	T2	Core biomarkers: Core 2	pT205, MTBR-243, non- phosphorylated tau fragments	Tau PET
	N	Biomarkers of non-specific processes involved in AD pathophysiology	NfL	Anatomic MR or CT, FDG PET
	I		GFAP	/
	V	Biomarkers of non-AD co-pathology	/	Anatomic infarction, WMH
	S		CSF αSyn-SAA	/

*Note*: the following biomarkers are included for conceptual purposes but have not undergone the same degree of testing as other Core biomarkers: P-tau231, p-tau205, MTBR-tau243, and non-phosphorylated tau.

**Table 2.** Study characteristics.

First Author (Year)	Title	Country	Study setting	Sample size	Age (as reported)	%Females (as reported)
Baiardi (2022)^17^	Diagnostic value of plasma p-tau181, NfL, and GFAP in a clinical setting cohort of prevalent neurodegenerative dementias	Italy	*Clinic.* Neuropathology Laboratory (NP-Lab) at the Institute of Neurological Science of Bologna, Italy.	376	Mean Age (SD): AD = 67.8 (9.3), HC = 61.7 (4.9), FTD = 62.9 (8.9); PSP = 69.2 (10.2); CBS = 71.3 (7.2); DLB = 73.7 (6.7); SNAP = 66.2 (9.5)	AD = 55.7, HC = 43.3, FTD = 57.6, PSP = 35.5, CBS = 62.1, DLB = 28.6, SNAP = 49.0
Cecchetti (2024)^18^	Diagnostic accuracy of automated Lumipulse plasma pTau-217 in Alzheimer's disease: a real-world study	Italy	*Clinic.* Neurology Unit of IRCCS San Raffaele Scientific Institute	98	Mean ± SD (min-max): AD-DEM = 70.9 ± 7.32 (53.30–83.78); AD-MCI = 73.07 ± 6.84 (53.26–84.04); NonAD-DEM = 71.17 ± 7.33 (56.34–80.73); NonAD-MCI = 69.51 ± 8.24 (51.54–83.56)	AD-DEM = 44.4, AD-MCI = 43.5, NonAD-DEM = 60.0, NonAD-MCI = 45.5
Chen (2024)^19^	Diagnostic value of isolated plasma biomarkers and its combination in neurodegenerative dementias: A multicenter cohort study	China	*4 Cohorts.* Zhejiang University School of Medicine (First Affiliated Hospital, Second Affiliated Hospital, Sir Run Run Shaw Hospital, and Affiliated Zhejiang Hospital)	112	Mean ± SD: Total = 64.4 ± 10.0, HC = 65.7 ± 9.1, AD = 62.8 ± 10.7, FTD = 64.3 ± 9.00, PSP = 71.8 ± 5.9	Overall = 51.7, HC = 50.0, AD = 48.4, FTD = 62.5, PSP = 58.3
Kivisäkk (2023)^20^	Plasma biomarkers for diagnosis of Alzheimer's disease and prediction of cognitive decline in individuals with mild cognitive impairment	USA	*Cohort.* MADRC- LC study, a longitudinal observational study of cognitive aging, AD, and AD-related disorders.	238 in diagnostic sample [Total N = 307]	Mean ± SD: CN = 72.6 ± 10.4, AD = 74.2 ± 10.6, OND = 69.4 ± 10.9	CN = 56.7, AD = 47.4, OND = 45.3
Lopes das Neves (2023)^26^	Serum Neurofilament Light Chain in the Diagnostic Evaluation of Patients with Cognitive Symptoms in the Neurological Consultation of a Tertiary Center	Portugal	*Clinic.* Dementia consultation service.	365	CTRs = 65.2 (±10.3); MCI = 67.2 (±9.1); AD = 64.0 (±6.4); FTD = 62.9 (±6.8) years	CTRs = 58.8, MCI data is incorrect in Table S1, AD = 60.5, FTD = 45.9
Oeckl (2019)^24^	Glial Fibrillary Acidic Protein in Serum is Increased in Alzheimer's Disease and Correlates with Cognitive Impairment	Germany	*Clinic.* Recruitment was done at Ulm University Hospital, Department of Neurology, LMU Munich, TU Munich, and University of Erlangen-Nuremberg.	127	median age (IQR): controls = 66 (57–74), PD = 73 (64–76), bvFTD = 64 (56–71), AD = 71 (67–78), DLB/PDD = 74 (70–76)	Overall = 39.4, Controls = 26.5, PD = 18.2, bvFTD = 42.9, AD = 67.9, DLB/PDD = 26.3
Oeckl (2022)^25^	Serum GFAP differentiates Alzheimer's disease from frontotemporal dementia and predicts MCI-­ to-dementia conversion	Portugal, Netherlands and Germany	*Clinic.* Patients recruited from University Hospital of Coimbra (Portugal), Radboud University Medical Center (Netherlands), the Technical University of Munich (Germany) and Ulm University Hospital (Germany).	610	median age (IQR): controls = 63 (57–69), bvFTD = 62 (57–68); MCI-AD = 71 (64–74), AD dementia = 69 (62–76)	Overall = 54.1, Controls = 48.8, bvFTD = 45.7, MCI-AD = 57.7, AD dementia = 60.4
Palmqvist (2020)^21^	Discriminative accuracy of plasma phospho-tau217 for Alzheimer disease vs. other neurodegenerative disorders	Sweden	*Cohort.* Subjects were recruited from the BioFINDER study.	699	median (IQR) age: CU =66.6 (55.3–76.1), MCI = 72.2 (65.3–75.9), AD dementia = 74.2 (70.4–78.1), other NDDs = 72.4 (64.0–76.5)	CU = 54.8, MCI = 44.9, AD dementia = 52.1, other NDDs = 48.5
Sarto (2023)^22^	Diagnostic Performance and Clinical Applicability of Blood-Based Biomarkers in a Prospective Memory Clinic Cohort	Spain	*Clinic.* Alzheimer's Disease and Other Cognitive Disorders Unit, Hospital Clınic, Barcelona, Spain.	268 in AD biomarker subcohort [Total N = 385]	mean (SD) age: CU = 61.7 (8.2), overall clinical population = 66.7 (7.5), SCD = 66.9 (5.4), MCI = 67.1 (7.4), AD dementia = 66.2 (6), LBD = 68.5 (6.7), FTD = 66.2 (9.4)	CU = 78, Overall clinical population = 53, SCD = 62, MCI = 55, AD dementia = 62, LBD = 30, FTD = 46
Shen (2023)^23^	Plasma Glial Fibrillary Acidic Protein in the Alzheimer Disease Continuum: Relationship to Other Biomarkers, Differential Diagnosis, and Prediction of Clinical Progression	China	*Clinic and cohort.* Subjects recruited from the Memory Clinic of the Huashan Hospital of Fudan University (Shanghai) and the Chinese Alzheimer Biomarker and LifestylE (CABLE) study	700	mean (SD) age: CN = 60 (9), MCI 64 (11), AD = 59 (8), PCA = 56 (5), PPA = 61 (8), bvFTD = 59 (9), FTD-MND 61 (7), PSP = 66 (7), VaD = 65 (8), DLB = 68 (9), MSA = 59 (8), PD = 64 (10), SCA = 46 (12), ALS = 51 (20)	CN = 55.9, MCI = 51.8, AD = 59.6, PCA = 53.8, PPA = 62.8, bvFTD = 50.0, FTD-MND = 71.4, PSP = 41.2, VaD = 28.9, DLB = 46.4, MSA = 44, PD = 45, SCA = 50.0, ALS = 50.0
Thijssen (2021)^28^	Association of Plasma P-tau217 and P tau181 with clinical phenotype, neuropathology, and imaging markers in Alzheimer's disease and frontotemporal lobar degeneration: a retrospective diagnostic performance study	USA	*Clinic.* Recruitment was done from the University of California San Francisco (UCSF) Memory and Aging Center, an NIA ADRC, and from the Advancing Research and Treatment for Frontotemporal Lobar Degeneration (ARTFL) consortium	593	Mean age (SD): Overall = 64.3 (13), CN = 60.9 (18), MCI = 65.5 (13), ADclin = 65.3 (10), lvPPA = 63.1 (9), PCA = 57.6 (11), CBS = 67.3 (8), PSP = 68.5 (7), bvFTD = 61.2 (10), nfvPPA = 69.8 (7), svPPA = 70.0 (7), DLB = 69.3 (6), TES = 63.2 (13)	Overall = 49.7, CN = 53.4, MCI = 44.4, ADclin = 56.9, lvPPA = 53.3, PCA = 100 (n = 2), CBS = 54.4, PSP = 54.1, bvFTD = 40.3, nfvPPA = 46.9, svPPA = 59.3, DLB = 35.7, TES = 0 (n = 13)
Thijssen (2022)^29^	Differential diagnostic performance of a panel of plasma biomarkers for different types of dementia	Netherlands	*Cohort.* Patients from both Cohort 1 and 2 were recruited from the Amsterdam Dementia Cohort.	160 (Cohort 1) + 152 (Cohort 2)	Median age (IQR): Cohort 1: Controls = 56 (53–59), AD = 58 (55–59), FTD = 64 (61–70), DLB = 70 (62–74), Overall = 59 (56–66); Cohort 2: Controls = 63 (59–66), AD = 63 (59–67), FTD = 63 (59–67), DLB = 67 (64–69), Overall = 64 (60–68)	Cohort 1: Controls = 50, AD = 50, FTD = 55, DLB = 30, Overall = 46; Cohort 2: Controls = 53, AD = 53, FTD = 53, DLB = 8, Overall = 41
Vrillon (2023)^27^	Comparison of CSF and plasma NfL and pNfH for Alzheimer's disease diagnosis: a memory clinic study	France	*Clinic.* Patients recruited from a tertiary memory center—the Center of Cognitive Neurology, at the University Hospital Lariboisière, Paris.	188	Mean age (SD): Controls = 63.4 (10.1), non-AD MCI = 68.7 (9.7), AD-MCI = 72.1 (7.4), AD dementia = 71.8 (8.4), non-AD dementia = 67.4 (7.6)	Controls = 68, Non-AD MCI = 66, AD-MCI = 53, AD dementia = 64, non-AD dementia = 54

**Table 3.** Diagnostic criteria and blood biomarkers.

First author (Year)	AD clinical diagnostic criteria [as reported]	AD biological diagnostic criteria (CSF/PET/Both)	Blood biomarkers Tested	Assay and/or Platform	Other biomarkers tested	Key outcomes
Baiardi (2022)^17^	Consensus clinical diagnosis according to 2011 NIA-AA criteria (McKhann et al.) and Dubois et al. (2014)	CSF Aβ_42/40_ ratio < 0.65, p-tau/Aβ_42_ ratio > 0.08, and t-tau/Aβ_42_ ratio > 0.52 supported AD diagnosis.	GFAP, NfL	SiMOA NF-light advantage, SiMOA GFAP discovery, both on SiMOA SR-X analyzer platform	Plasma p-tau181	GFAP and NfL had AUCs >0.90 to distinguish between AD and HC, but AUC <0.90 for all differential diagnostic comparisons.
Cecchetti (2024)^18^	Clinical diagnosis of mild cognitive impairment (MCI) or dementia at discharge, along with a detailed clinical/neurological evaluation and neuropsychological assessment within 6 months from lumbar puncture	CSF Aβ_42_, Aβ_40_ and pTau-181 according to the following cut-offs: Aβ_42_ ≥ 500 ng/L; p-tau ≤60 ng/; p-tau/Aβ_42_ ≤ 0.12.	pTau217	Automated CLEIA on LUMIPULSE G600II system	Plasma Aβ_42_, Aβ_40_, pTau-181	Plasma pTau217 is a potential standalone diagnostic biomarker for AD, and differentiated AD from other dementias with an AUC of 0.97.
Chen (2024)^19^	DSM-IV + 2018 NIA-AA criteria (Jack et al.)	^18^F-florbetapir PET and/or CSF Aβ_42/40_. [for AD, n = 40 confirmed by CSF LP and n = 26 AD by PET]	GFAP, NfL	Human Neurology 4-Plex E assay kit (fpr plasma Aβ_42_, Aβ_40_, NfL, and GFAP) on automated SIMOA HD-X analyzer.	Plasma Aβ_42_, Aβ_40_, pTau-181	Neither NfL nor GFAP showed strong diagnostic / differential diagnostic performance as standalone biomarkers (all AUCs<0.85).
Kivisäkk (2023)^20^	Consensus diagnosis in line according to 2011 NIA-AA criteria for MCI and AD.	Intermediate/ high ADNC upon autopsy, [^11^C]Pittsburgh Compound-B amyloid PET imaging, and/or CSF AD biomarkers	GFAP, NfL	MSD S-PLEX for NfL and GFAP	Plasma pTau181, tTau	In combination with a base model including age, sex and APOE status, the AUC of NfL and GFAP in differentiating AD from CN, and AD from OND were between 0.80–0.85.
Lopes das Neves (2023)^26^	Clinical diagnosis according to 2011 NIA-AA criteria (McKhann et al.)	CSF Aβ_42_ or Aβ_42_/Aβ_40_ ratio, total tau (t-tau), and p-tau (97.9% cases) or genetic confirmation	NfL	NF-light Advantage kit (SR-X platform, Quanterix).	CSF NfL	Serum NfL distinguised AD from CTRs and FTD with similar AUC (0.75. 0.74 respectively), and can be used as a proxy for CSF NfL
Oeckl (2019)^24^	Participants met criteria for “probable or possible AD dementia with evidence of the AD pathophysiological process” (i.e., CSF Tau and Aβ_42_), in line with 2011 NIA-AA criteria (McKhann et al.)	CSF Tau and Aβ42	GFAP	SiMOA GFAP Discovery	CSF GFAP, CSF Tau and CSF Aβ_42_	Serum GFAP could discriminate between AD controls at a cut-off of 219 pg/mL (AUC = 0.91) and between AD and bvFTD at a cut off of 289 pg/mL (AUC = 0.85).
Oeckl (2022)^25^	Biomarker-supported diagnosis of AD dementia and AD MCI according to 2011 NIA-AA criteria (McKhann et al.) and Albert et al. (2011)	CSF Aβ_42_, total tau and pTau181	GFAP	SiMOA GFAP Discovery	Serum NfL, Serum pTau181	Patients with AD dementia could be separated from controls with an of AUC 0.87, and AD dementia could be differentiated from bvFTD with an AUC of 0.81. Cut-offs for the diagnostic (AD vs. HC) and differential diagnostic (AD vs. bvFTD) were similar (245 pg/mL and 246 pg/ml respectively).
Palmqvist (2020)^21^	DSM-V AD criteria	CSF Aβ_42_/Aβ_40_ with a cutoff of <0.752	pTau217	Lilly plasma pTau217, MSD platform	plasma pTau181, NfL (as reference biomarker), hippocampal volume, AD signature cortical thickness, CSF pTau217, pTau181, Tau-PET.	Plasma pTau217 has excellent discriminatory power between AD dementia and other NDDs, irrespective of Aβ status.
Sarto (2023)^22^	Clinical diagnostic work-up comprised neurologic assessment, neuropsychological testing, and structural neuroimaging.	CSF Aβ_1−42_, Aβ_1−42_/Aβ_1−40_ ratio	GFAP, NfL	Quanterix Simoa Neurology 4-Plex A (for t-tau, GFAP, NfL, and UCH-L1)	Plasma t-tau, UCH-L1, p-tau181	Plasma GFAP and NfL show varied diagnostic performance. Overall, GFAP performed better than NfL in discriminating AD from CU, SND and LBD, as compared to NfL. Although NfL performed better in discriminating AD from FTD, its performance was moderate.
Shen (2023)^23^	AD clinical diagnosis in line with 2011 NIA-AA “dementia due to AD” criteria (McKhann et al.)	CSF Aβ_1−42_ with an optimal threshold of <194.5 pg/mL. (+ Amyloid PET for subset)	GFAP [NfL as reference biomarker]	Quanterix SIMOA Neurology 4-Plex E (for GFAP, Aβ_1−40_, Aβ_1−42_, and NfL)	Plasma Aβ_1–40_, Aβ_1–42_, p-tau181, CSF GFAP	Plasma GFAP showed high diagnostic accuracy in discriminating AD from CN (AUC 0.973), and other non-AD dementias, most notably ALS and SCA (AUCs 0.989 and 0.979 respectively).
Thijssen (2021)^28^	Clinical diagnosis with standardized evaluation involving demographic features, neurological, neuropsychological, and functional assessment with informant interview. Severity measured by Clinical Dementia Rating instrumental global and sum of boxes scores, Functional Activities Questionnaire and Schwab and England Activities of Daily Living scale	Aβ-PET (n = 360), autopsy (n = 83) or genetic biomarker-confirmation	pTau217, NfL	Homebrew/ commercial kit on Quanterix HD-1 analyzer. Plasna pTau217: MSD, NfL: SiMOA	Plasma pTau181	Plasma pTau217 outperforms NfL in AD vs. CN and AD vs. FTLD diagnostic comparisons.
Thijssen (2022)^29^	Neurological, physical, and neuropsychological evaluations, with multidisciplinary consensus diagnoses based on clinical criteria from 2018 NIA-AA criteria Jack et al.	CSF Aβ_1−42_, with cutoff of 813 pg/mL for Innotest ELISA, and 1000 pg/mL for the Elecsys assay.	GFAP, NfL	Pre-commercial/ commercial versions of Neurology 4-plex E kit (for GFAP, NfL, Aβ_1−42_ and Aβ_1−40_)	Plasma Aβ_1−42_, Aβ_1−40_, Aβ_1−42/1−40_ ratio, pTau181	NfL and GFAP have moderate performance in differential diagnosis of AD. In both cohorts, both biomarkers distinguished AD from FTD better than AD from DLB.
Vrillon (2023)^27^	Diagnosis for AD-MCI in line with by Albert et al. (2011) and for AD by 2011 NIA-AA critieria (McKhann et al.)	CSF Aβ_42_/Aβ_40_, p-tau and t-tau, in line with the cut-offs: Aβ_42_/Aβ_40_ < 0.061, p-tau > 61 ng/L and t-tau > 479 ng/L	NfL	Commercial kit by­ Quanterix^®^ on the SIMOA platform.	CSF NfL, CSF NfH, plasma NfH	Plasma NfL is a potential proxy candidate for CSF NfL in an unselected and consecutive memory clinic population.

**Table 4.** Outcomes.

First author (Year)	Diagnostic comparisons	Blood biomarkers tested	AUC (95% CI) [as reported]
Baiardi (2022)^17^	AD vs. HC	**GFAP**	**0**.**939**
	AD vs. HC	**NfL**	**0**.**908**
	AD vs. OND (PSP + CBS + DLB + FTD)	GFAP	0.703
	AD vs. FTD	GFAP	0.818
	AD vs. FTD	NfL	0.791
	AD vs. PSP	GFAP	0.765
	AD vs. CBS	GFAP	0.615
	AD vs. DLB	GFAP	0.578
Cecchetti (2024)^18^	AD-DEM vs. nonAD-DEM	**pTau217**	**0**.**973**
Chen (2024)^19^	AD vs. HC	GFAP	0.844
	AD vs. HC	NfL	0.833
	AD-dem vs. non-AD dem (PSP + FTD)	GFAP	0.579
	AD-dem vs. non-AD dem (PSP + FTD)	NfL	0.704
Kivisäkk (2023)^20^	AD vs. CN	Base model + GFAP	0.81
	AD vs. CN	Base model + NfL	0.85
	AD vs. OND	Base model + GFAP	0.80
	AD vs. OND	Base model + NfL	0.81
Lopes das Neves (2023)^26^	CTRs vs. AD	NfL	0.75 (CI?)
	AD vs. FTD	NfL	0.74 (CI?)
Oeckl (2019)^24^	AD vs. Controls	**GFAP**	**0**.**91**
	AD vs. bvFTD	GFAP	0.85
Oeckl (2022)^25^	AD dementia vs. Controls	GFAP	0.87
	AD dementia vs. bvFTD	GFAP	0.81
	*MCI-AD vs. Controls*	*GFAP*	*0*.*76*
	*MCI-AD vs. bvFTD*	*GFAP*	*0*.*75*
Palmqvist (2020)^21^	AD dementia vs. all other NDDs	**pTau217**	**0**.**96**
	AD dementia vs. bvFTD/PPA	**pTau217**	**0**.**92**
	AD dementia vs. VaD	**pTau217**	**0**.**97**
	AD dementia vs. PD/PDD/MSA	**pTau217**	**0**.**97**
	AD dementia vs. PSP/ CBS	**pTau217**	**0**.**96**
	*AD dementia vs. Aβ- controls*	***pTau217***	***0***. ***98***
	*AD dementia vs. Aβ- MCI*	***pTau217***	***0***. ***97***
	*AD dementia vs. All Aβ- other NDDs,*	***pTau217***	***0***. ***96***
	*AD dementia vs. All Aβ+ other NDDs*	***pTau217***	***0***. ***93***
Sarto (2023)^22^ [AD biomarker sub-cohort only]	CU vs. AD	GFAP	0.85
	CU vs. AD	NfL	0.75
	AD vs. SND	**GFAP**	**0**.**92**
	AD vs. SND	NfL	0.81
	AD vs. LBD	GFAP	0.76
	AD vs. LBD	NfL	0.55
	AD vs. FTD	GFAP	0.65
	AD vs. FTD	NfL	0.79
Shen (2023)^23^	AD vs. CN	**GFAP**	**0**.**973**
	AD vs. VaD	GFAP	0.859
	AD vs. FTLD	GFAP	0.856
	AD vs. ASRD	GFAP	0.799
	AD vs. ALS	**GFAP**	**0**.**989**
	AD vs. SCA	**GFAP**	**0**.**979**
Thijssen (2021)^28^	AD vs. Controls	**pTau217**	**0**.**98**
	AD vs. Controls	NfL	0.67
	AD vs. FTLD	**pTau217**	**0**.**93**
	AD vs. FTLD	NfL	0.82
Thijssen (2022)^29^	AD vs. FTD (Cohort 1)	GFAP	0.81
	AD vs. FTD (Cohort 1)	NfL	0.79
	AD vs. DLB (Cohort 1)	GFAP	0.69
	AD vs. DLB (Cohort 1)	NfL	0.50
	AD vs. FTD (Cohort 2)	GFAP	0.71
	AD vs. FTD (Cohort 2)	NfL	0.78
	AD vs. DLB (Cohort 2)	GFAP	0.65
	AD vs. DLB (Cohort 2)	NfL	0.63
Vrillon (2023)^27^	AD dem vs. controls	NfL	0.88
	non-AD dem vs. AD dem	NfL	0.75
	AD dementia vs. FTD	NfL	0.75
	AD dementia vs. DLB	NfL	0.69
	***AD-MCI vs. controls***	NfL	0.85
	***AD-MCI vs. non-AD MCI***	NfL	0.65

Current evidence suggests that these biomarkers are clinically relevant: plasma pTau217 has shown diagnostic performance superior to other phosphorylated tau isoforms in both preclinical and clinical stages of AD dementia.^6–8^ Additionally, both GFAP and NfL are known to be elevated in AD brains,^9^ and CSF,^10–13^ and have been shown to differentiate AD from other dementias.^14–16^ Further, blood biomarkers have arguably the most utility as screening and diagnostic tools in clinical settings, where patients present with a variety of co-pathologies. In such contexts, a combination of AD-specific biomarkers like pTau217, and non-specific biomarkers of associated neurodegenerative and inflammatory pathological processes (NfL and GFAP, respectively), could prove to have immense clinical significance.

Considering the above, this systematic review evaluates the diagnostic and differential diagnostic utility of pTau217, GFAP, and NfL in AD. Specifically, this review focuses on studies conducted in real-world clinical populations (e.g., memory clinics), where blood biomarker roll-out is most likely, and will offer an accessible alternative to present diagnostic techniques, that can be scaled to diverse, under-resourced settings including LMICs.

## Methods

### Search and screening

**Figure 1.**
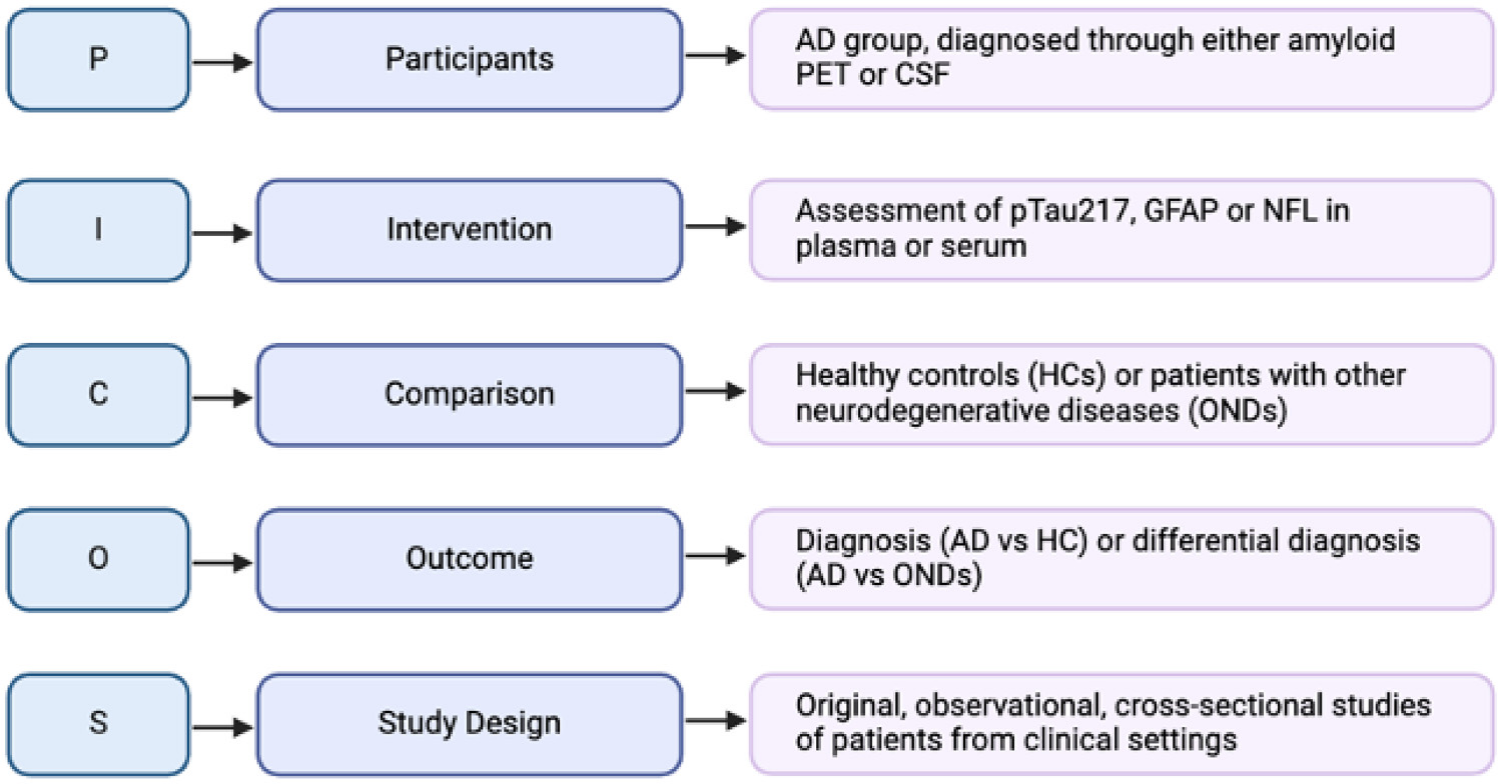
Review design according to PICOS framework.

The systematic review was undertaken in accordance with the PRISMA guidelines, designed according to the PICOS framework (Figure 1), and was registered on the International Prospective Register of Systematic Reviews (PROSPERO: https://www.crd.york.ac.uk/PROSPERO/view/CRD42024524962/), registration number CRD42024524962.

The systematic search was conducted in two phases: the first phase included studies published between 1 January 2019 and 31 December 2023, and the second was an update search carried out from 31 December 2023 to 27 January 2025. Both searches included publications across multiple databases (PubMed, EMBASE, Global Health, Journals@OVID and PsycINFO) and were limited to articles published in English in peer-reviewed journals.

Articles were selected as per the specific inclusion criteria: (1) the outcome of the article was to assess diagnostic or differential diagnostic ability of a biomarker of interest, or any combinations of the biomarkers of interest, (2) the population was partially or completely recruited from a clinical setting (for instance, a memory clinic or neurology department in a hospital), (3) the study had a clearly defined AD patient group, in which participants had both a clinical diagnosis consistent with AD (i.e., etiological confirmation) and biological confirmation of AD pathology via CSF or PET biomarkers.

Articles were excluded if (1) the study design and outcomes were in line with risk prediction or prognosis only, without any diagnostic element, (2) the population was exclusively cognitively normal, consisted of individuals with memory complaints without an AD-confirmed group, or was subject to strict recruitment criteria (for instance, a cohort study which categorically excluded patients with AD or other dementia diagnoses), (3) the population represented genetic variants of AD alone, (4) the biomarker of interest was measured in combination with a biomarker other than GFAP, NfL or pTau217, or was measured using a non-scalable method (5) the biomarker of interest was used as a reference, to assess efficacy of another biomarker or an intervention, (6) the article was associative, or aimed at elucidating underlying mechanisms of disease, neuropathology, (7) the study was carried out in a biofluid other than blood, or not in a human population (e.g., cell lines, mice), (8) any grey literature (conference abstract, poster presentation, book chapter, theses, etc.), meta-analyses or reviews.

Search results were imported into Rayyan, and duplicates were removed. Title- and abstract- screening, and subsequent full text screening were done independently by SS and LM. Any disagreements during the screening process were resolved by a consensus between SS, LM, SB, and VR.

### Quality assessment and data extraction

Quality assessment of all included articles was conducted using the QADAS-2 tool by SS and LM. Signaling questions in all 4 domains of the QADAS questionnaire (patient selection, index test, reference standard and flow and timing) were tailored to ensure review-specific assessment of bias. Relevant data on study characteristics, biomarker- and diagnostic information, and outcomes were extracted by SS and LM. Narrative synthesis was conducted by SS.

## Results

### Study selection

**Figure 2.**
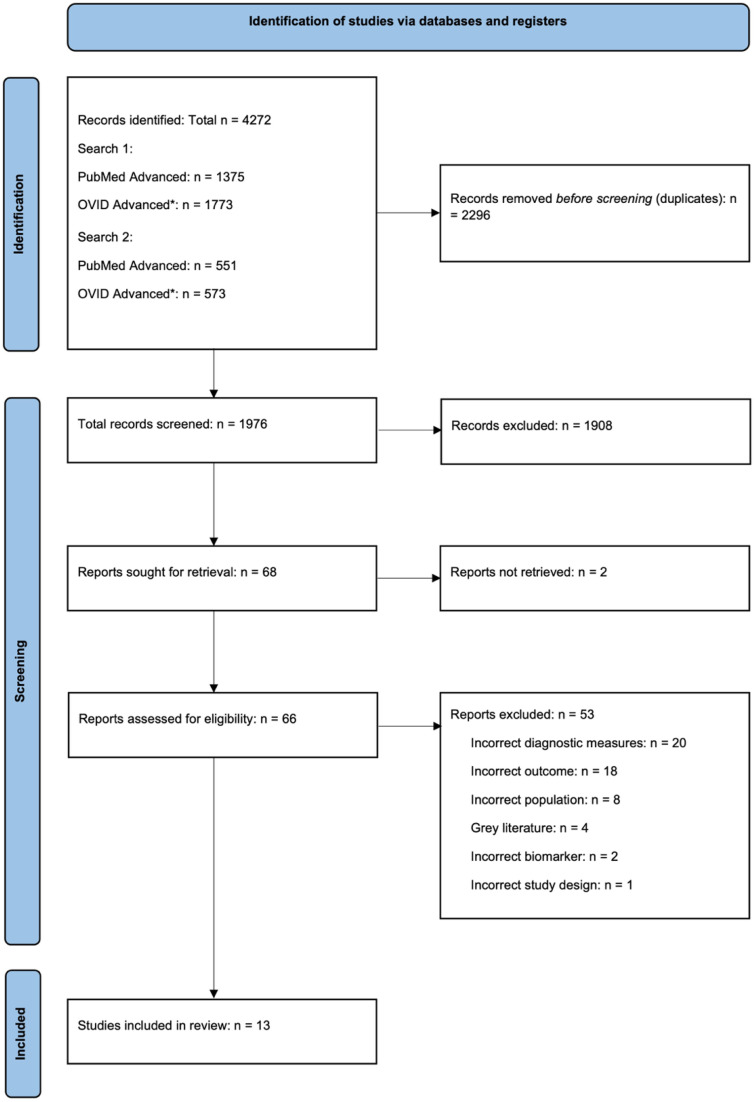
PRISMA flowchart for Systematic Review search and screening. *OVID advanced search included the following databases: Journals @ Ovid, Embase, Global health, PsycINFO.

The search identified 4272 articles across five databases (PubMed, Journals @ OVID, Embase, Global Health, and PsycINFO) (Figure 2). After eliminating 2296 duplicates, 1976 articles remained for title- and abstract screening. At this stage, 1908 articles were excluded as they were irrelevant to the review question, leaving 68 articles for full-text review. Of these, the full-text version of 2 reports was not available, and 49 reports were excluded for having ineligible diagnostic measures, biomarkers, outcomes, patient populations, or study design, and 4 were excluded as they were poster presentations or conference abstracts. Consequently, 13 articles were included in the review.^17–29^

### Study characteristics

Of the thirteen included studies, eight studies were conducted directly in specialist memory clinics or hospital neurology departments, 4 analyzed data from clinical cohorts, and 1 included patient data collected partially from cohorts, and partly from clinic (Table 2). Four studies explicitly mentioned consecutive patient recruitment, enhancing representation of real-world clinical settings by minimizing selection bias.

A total of 4686 participants were studied across all included articles. In some studies, only specific sub-cohorts relevant to the research question were assessed. Sample sizes were varied across studies, ranging from 98 in Cecchetti et al. (2024)^18^ to 700 in Shen et al. (2023).^23^ The average/ median age of the AD group was higher than or equal to that of the cognitively unimpaired (CU) group in nine cases and lower in three cases. One study (Cecchetti et al.^18^) lacked a CU group. All studies had a fair representation of both males and females, assessed differences in age between diagnostic groups, and controlled for these factors in analyses if significant effects were found. There was some variability in demographic reporting: 8 studies recorded age as mean (SD) while 4 studies reported median (IQR).

The thirteen studies that met the inclusion criteria of the review represented nine countries. Three studies reported ethnicity: Kivisakk et al. (2023)^20^ reported 93.7% non-Hispanic whites, Thijssen et al. (2021)^28^ reported an 85% white population, and Shen et al. (2023)^23^ reported exclusively Chinese participants.

### Diagnostic criteria and blood biomarkers

There was substantial variability in the methods used to confirm AD diagnosis was observed, both clinically and biologically (Table 3). Six studies established clinical etiology according to the 2011 NIA-AA criteria, two according to the 2018 NIA-AA criteria, two studies used the DSM criteria, and three studies did not mention any specific guidelines. Despite 11 of 13 selected studies using CSF biomarkers to confirm AD diagnosis, the specific biomarkers varied ranged from only amyloid markers (CSF Aβ_1−42_, Aβ_1−42_/Aβ_1−40_ ratio) to a combination of amyloid and tau markers (such as CSF total tau, p-tau, p-tau181), and hybrid ratios (Aβ_42_/p-tau ratio). Amyloid PET was utilized in four studies, and genetic confirmation, and pathological diagnosis on autopsy were employed in addition to CSF/PET biomarkers in some cases.

Six studies evaluated more than one blood biomarker of interest, and no study evaluated all three biomarkers. All selected articles assessed other biomarkers, beyond the scope of this review. These were most commonly plasma pTau181 and amyloid markers in both plasma and CSF.

The diagnostic performance of pTau217 was assessed in three studies, all using different assays and platforms for analysis (Automated CLEIA on LUMIPULSE G600II system, Lilly plasma pTau217 on the MSD platform, commercial/homebrew kit on the Quanterix HD-1 analyzer). Along a similar vein, the eight studies that measured GFAP and NfL also used various assays for biomarker measurement. Individual measurements of NfL were either reported using the NF-light advantage kit, or a “commercial kit by Quanterix”,^27^ or a “homebrew or commercially available kit”.^28^ Additionally, four studies used multiplex assays for the simultaneous measurement of plasma GFAP and NfL. Kivisakk et al. (2023)^20^ used an ultrasensitive prototype S-PLEX assay on the MSD platform, Thijssen et al. (2022)^29^ and Shen et al. (2023)^23^ used the Neurology 4-plex E kit to for simultaneous measurement of GFAP, NfL, Aβ_1−42_ and Aβ_1−40_, and Sarto et al. (2023)^22^ used the Neurology 4-plex A kit to analyze GFAP, NfL, UCH-L1 and t-tau.

Overall, several assays were used to measure all biomarkers on both MSD and SiMOA platforms. Different versions of the SiMOA platform were used in different studies (e.g., SR-X, HD-1), leading to less standardization while measuring the same biomarker. Taken together, this reflects heterogeneity in biomarker measurement techniques.

No study measured the biomarkers relevant to the review in a panel with one another. Combinations of biomarkers of interest with those that are not within the scope of this review were excluded to maintain consistency in the relevant biomarkers.

### Outcomes

Eleven out of the selected thirteen studies reported both diagnostic (AD versus CU) and differential diagnostic (AD versus other neurodegenerative disease(s)) comparisons, two reported differential diagnosis alone (Table 4). Studies whose outcome was solely the detection of amyloid positivity were systematically excluded from this review to maintain focus on the dementia phase of the AD continuum.

Of the three biomarkers of interest, only pTau217 consistently had an accuracy of >0.90 across all studies, and all twelve diagnostic comparisons. Although GFAP generally showed stronger diagnostic performance than NfL, especially in distinguishing AD from, the performance of both these biomarkers was moderate and varied. Of the thirty diagnostic comparisons in which GFAP was evaluated, six had an AUC > 0.90, sixteen had an AUC >0.75–0.90, and eight reported AUC < 0.75. Twenty-four comparisons were investigated for NfL, of which only one had an AUC > 0.90.

## Discussion

This systematic review evaluates the utility of three blood-based biomarkers, p-tau217, GFAP, and NfL, in the diagnosis and differential diagnosis of AD dementia in patients recruited from clinical settings, such as memory clinics and hospital neurology departments. The development of highly sensitive blood-based tests to detect and diagnose AD presents a highly resource-efficient, non-invasive and accessible alternative to traditional CSF and PET neuroimaging techniques, that are expensive and non-scalable.^30^ These biomarkers were recognized in the Alzheimer's Association Workgroup's recent “Revised Criteria for Diagnosis and Staging of Alzheimer's disease”, which emphasized their contribution in recent AD dementia research, and highlighted the likelihood of their regulatory approval imminently.^4^ The revised criteria will play an important role in laying the groundwork for rollout of commercial plasma biomarker tests in coming years, particularly in clinical services, given their likely upscale given the advent of disease modifying therapies.^31,32^ In light of this, the current review is timely in synthesizing the current literature, pointing out gaps in the literature, and laying the groundwork for future studies.

One of the key recommendations of the Alzheimer's Association's 2024 *Revised Criteria* demonstrate an accuracy of >90%, or equivalent to its CSF counterpart. All studies assessing diagnostic utility of plasma p-tau217 are consistent with this recommendation. One of these studies, conducted by Thijssen et al. (2021),^28^ also demonstrated the superior performance of p-tau217 as compared to NfL across all diagnostic comparisons. This is expected as NfL is a non-specific biomarker of neurodegeneration, especially in disorders of cognitive dysfunction.^12,33,34^ While the combined performance of p-tau217 and NfL was not explored, individual evaluation of NfL was investigated. Although two independent evaluations of NfL reported moderate diagnostic accuracy, both demonstrated that the diagnostic performance of plasma NfL was equivalent to its CSF counterpart, suggesting increased clinical utility and scalability.^26,27^ Similar findings have been reported in longitudinal studies of a cohort of non-demented participants (cognitively normal, or with mild cognitive impairment).^35^ However, a meta-analysis evaluating the overall pooled correlation coefficient estimate between NfL in both these biofluids reported only moderate correlations.^36^

In contrast to NfL, GFAP showed >90% accuracy in AD diagnosis^23,24^ and differential diagnosis, specifically against non-AD pathology,^22^ as well as amyotrophic lateral sclerosis and spinocerebellar ataxia.^23^ Notably, the study by Shen et al.^23^ is the only selected study to report findings from an LMIC and could provide valuable insights into the global applicability of GFAP in clinical settings. In the four studies that assessed accuracy of both NfL and GFAP, GFAP outperformed NfL in most diagnostic comparisons. Analogously, GFAP outperformed NfL in predicting AD incidence in population-based community cohort representing AD and other neurodegenerative disorders followed for 17 years, suggesting similar trends in studies aimed at risk prediction and prognosis.^37^

The major strengths of this review are the specific focus on clinical populations, evaluation of biomarkers of high clinical relevance, and the validation of blood biomarkers against gold-standard diagnostic methods (PET or CSF). However, some of the initial hypotheses of the review were not met. For instance, combinations or panels of the selected biomarkers only were not performed in any of the included studies. This is surprising for a few reasons. First, it was anticipated that the inclusion of differential diagnosis as an outcome would prompt the selection of articles that looked at AD-specific (p-tau217), along with non-specific (GFAP and NfL) biomarkers as these are expressed differentially in neurodegenerative conditions and might, intuitively, provide a more holistic insight into heterogenous pathologies represented in real-world, memory clinic settings.^38^ For instance, NfL has been shown to identify dementia in Down syndrome and among psychiatric conditions, and even between demented and non-demented Parkinson's disease patients.^39,40^ This gap might, in part, be due to the exclusion of comparisons of a selected biomarker with another biomarker like p-tau181 or plasma Aβ_42/40_. This biomarker selection trade-off was considered during the conceptualization phase of the review, and was justified since, (a) p-tau217 has shown superior performance to other p-tau isoforms,^8^^41–44^ (b) plasma Aβ_42/40_ assays have shown limited robustness and diagnostic range,^45,46^ and (c) an intention to keep biomarker selection consistent with the *Revised Criteria* was a major driving force for the current review.

A similar trade-off was also considered while excluding data from exclusively cognitively healthy, pre-clinical populations, or those from population-based- or community- cohorts. Importantly, as community- or population- cohort enrolment hinges on voluntary participation, often, at-risk groups are inherently excluded, and this limits generalizability in clinical populations. Yet, as much of clinical research has utilized data from cohorts and health registries, articles were included if the cohort mentioned at least partial recruitment from clinical services. Notably, through the screening process, it was observed that cohorts including minority- or non-White ethnicities recruited exclusively through community outreach initiatives and not through clinical services, suggesting a lack of healthcare utilization and access for ethnic minority groups.

Eleven studies represented patient populations from World-Bank designated high income countries: France, Germany, Italy, Netherlands, Portugal, Sweden, Spain, and USA, and two studies presented data from Chinese populations. Adjacently, only three included articles reported ethnicity, of which two reported >85% White participants. Geographical representation was also limited, with only one study being conducted in a non-High-income country. This limitation reflects disparities in resources and infrastructure, which perpetuate the use of clinical judgement alone, without biomarker-backed diagnoses, in resource-deficient settings.^47,48^ This creates a paradox: while the use of blood biomarker tests might bridge this disparity, their validation requires data from the very populations that currently lack access to currently-approved, gold standard methods. Efforts to improve inclusion and ethnicity reporting in clinical cohorts must be emphasized in future research. In addition to ethnicity, many analyses in the included article failed to consider social determinants of health, such as social contact, socioeconomic status, and discrimination, that have increasingly shown an association with dementia outcomes.^49,50^ This omission also reduces the applicability and relevance of the above findings to historically marginalized groups. Additionally, it is important to note that while the review's exclusive focus on cross-sectional studies in memory clinic settings provides increased relevance in routine clinical practice, it restricts the scope of blood biomarker utility in earlier phases of the AD continuum, which has been widely demonstrated.^43,51^

In this review, plasma pTau217 demonstrated the highest diagnostic accuracy for AD. Both GFAP and NfL displayed moderate results, that varied depending on the cohort, analytical platform, and diagnostic comparison. Despite the growing body of evidence showcasing the effectiveness and advantages of plasma biomarkers, significant gaps in the literature persist, especially in terms of representation of real-world, diverse, clinical settings, and the absence of contextual variables. These issues need to especially considered in the context of blood biomarkers, given that they provide a highly accessible, globally scalable, and resource-effective alternative for current diagnostic methods.

## Supplemental Material

sj-docx-1-alz-10.1177_13872877251408510 - Supplemental material for Blood biomarkers for diagnosis and differential diagnosis of Alzheimer's disease in real-world clinical populations: A systematic review Supplemental material, sj-docx-1-alz-10.1177_13872877251408510 for Blood biomarkers for diagnosis and differential diagnosis of Alzheimer's disease in real-world clinical populations: A systematic review by Shivani Suresh, Luciana Maffei, Sarah Bauermeister and Vanessa Raymont in Journal of Alzheimer's Disease
