# Supplementary material for: Blood biomarkers for diagnosis and differential diagnosis of Alzheimer's disease in real-world clinical populations: A systematic review
Source: J Alzheimers Dis. 2025 Dec 29;109(4):1574–89. doi: 10.1177/13872877251408510 (PMC12901672; doi:10.1177/13872877251408510)

**Supplemental Material**

**Blood biomarkers for diagnosis and differential diagnosis of Alzheimer’s disease in real-world clinical populations: A systematic review**

**Supplemental Figure 1.** Risk of Bias assessment for selected studies using the QADAS-2 tool.


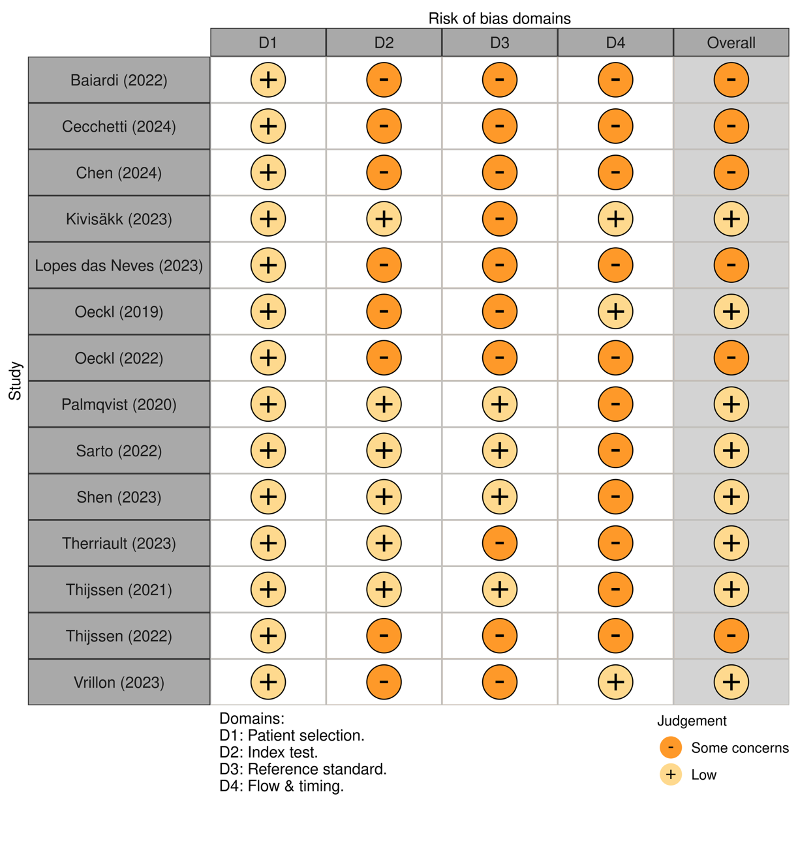

Supplement: sj-docx-1-alz-10.1177_13872877251408510 - Supplemental material for Blood biomarkers for diagnosis and differential diagnosis of Alzheimer's disease in real-world clinical populations: A systematic review [file sj-docx-1-alz-10.1177_13872877251408510.docx]
